# Complete mitogenome of the endangered and endemic Nicobar treeshrew (*Tupaia nicobarica*) and comparison with other Scandentians

**DOI:** 10.1038/s41598-022-04907-7

**Published:** 2022-01-18

**Authors:** Shantanu Kundu, Avas Pakrashi, Manokaran Kamalakannan, Devkant Singha, Kaomud Tyagi, Dhriti Banerjee, Chinnadurai Venkatraman, Vikas Kumar

**Affiliations:** 1 Centre for DNA Taxonomy, Molecular Systematics Division, Zoological Survey of India, Calcutta, 700053 India; 2 Mammal and Osteology Section, Zoological Survey of India, Calcutta, 700053 India

**Keywords:** DNA, Next-generation sequencing, Zoology, Genome, Genomics, Evolution, Molecular evolution, Phylogenetics, Taxonomy

## Abstract

The Nicobar treeshrew (*Tupaia nicobarica*) is an endangered small mammal endemic to the Nicobar Island of the Andaman Sea, India regarded as an alternative experimental animal model in biomedical research. The present study aimed to assemble the first mitochondrial genome of *T. nicobarica* to elucidate its phylogenetic position with respect to other Scandentians. The structure and variation of the novel mitochondrial genome were analyzed and compared with other Scandentians. The complete mitogenome (17,164 bp) encodes 37 genes, including 13 protein-coding genes (PCGs), 22 transfer RNA (tRNAs), two ribosomal RNA (rRNAs), and one control region (CR). Most of the genes were encoded on majority strand, except *nad6* and eight tRNAs. The nonsynonymous/synonymous ratio in all PCGs indicates strong negative selection among all Tupaiidae species. The comparative study of CRs revealed the occurrence of tandem repeats (CGTACA) found in *T. nicobarica*. The phylogenetic analyses (Maximum Likelihood and Bayesian Inference) showed distinct clustering of *T. nicobarica* with high branch supports and depict a substantial divergence time (12–19 MYA) from the ancestor lineage of Tupaiidae. The *16S rRNA* dataset corroborates the taxonomic rank of two subspecies of *T. nicobarica* from the Great and Little Nicobar Islands. In the future, whole nuclear genome sequencing is necessary to further improve our understanding of evolutionary relationships among treeshrews, and will have implications for biomedical research.

## Introduction

The world treeshrew account for 23 species under four genera (*Anathana*, *Dendrogale*, *Ptilocercus* and *Tupaia*) of two families (Tupaiidae and Ptilocercidae) which are distributed in South Asia, Southeast Asia, and Southwest China^1,2^. The mainland of India is known by two species, the Madras treeshrew, *Anathana ellioti* and the Northern treeshrew, *Tupaia belangeri*. However, the Nicobar treeshrew, *Tupaia nicobarica* is endemic to the Nicobar Islands^3^. This species is categorized as ‘Endangered’ species in the IUCN Red List of Threatened Species and listed under ‘Appendix II’ in the Convention on International Trade in Endangered Species of Wild Fauna and Flora (CITES)^4^. The treeshrews, including *T. nicobarica* are arboreal in nature and live in different forest types including scrub jungle, moist deciduous forests, and montane sholas. The members of *Anathana*, *Dendrogale*, and *Tupaia* are diurnal and solitary in habit; however the *Ptilocercus* species is nocturnal and live in family groups^4^.

The biogeography of India is mainly classified into two categories, the mainland and groups of islands (Lakshadweep and Andaman-Nicobar). The Andaman and Nicobar (AN) archipelago comprises 572 islands located on the Bay of Bengal^5^. The AN archipelago was formed due to collision between the Indian Plate and Eurasian Plate which commenced about 50 million years ago and continues today^6^. Due to the distance of the archipelago from the mainland and different diachronic processes, this group of islands accommodates numerous unique elements of biodiversity^7^. This archipelago is a trenchant biogeographic entity that can be a model for evolutionary studies^8,9^. The study on faunal diversity of AN archipelago has been started since nineteenth century, considering the large size or charismatic vertebrate fauna, including mammals, birds, and herpetofauna^10–12^. Due to the remoteness and inaccessibility throughout the year, most of the regions of AN archipelago are sparsely explored. Remarkably, these oceanic islands also provide a suitable habitat for many smaller mammals like, treeshrews (order Scandentia), shrews (order Eulipotyphla), and rodents (order Rodentia) due to their preferable spatial niche and carrying capacity^13^. Among them, the Great and Little Nicobar Islands are located about 1800 km east of mainland India, 600 km west of Thailand, 950 km north of Myanmar, and 180 km of Sumatra. On the basis of geographical distribution, two subspecies have been recognized, viz., *T. nicobarica nicobarica* from the Great Nicobar Island and *T. nicobarica surda* from the Little Nicobar Island^14^. However, the status of these subspecies has been endorsed pending further molecular studies^3,15,16^.

Remarkably, the zoonotic disease has been originated or transmitted through different mammalian species including treeshrews and cause life threatening to human beings throughout the globe^17,18^. Hence, the treeshrews species is evidenced to be a significant model for various human disorders like, depression, myopia, hepatitis B and C virus infections, and hepatocellular carcinoma^19,20^. The molecular study and genome sequencing has been demonstrated the genetic basis of signaling pathways in nervous and immune systems of the Chinese treeshrew and evidenced as a potential model for biomedical research^21^.

The characterizations of complete mitogenomes are widely used genomics approaches for systematics studies and evolutionary research of wider group of taxa including mammals^22–24^. So far, the complete mitogenome of five species, *Tupaia belangeri*, *Tupaia minor*, *Tupaia montana*, *Tupaia splendidula*, and *Tupaia tana* were generated from different geographical regions. The molecular studies were previously aimed to infer the phylogenetic and evolutionary relationship, genetic structure, and possible gene flow of Scandentia across their range distribution in Southeast Asia^25,26^. In addition, the partial mitochondrial genes (*12S rRNA*, *16S rRNA*, and *Cyt b*) were also utilized to elucidate the phylogenetic position and diversification of Tupaiidae species including *T. nicobarica*^15,27,28^. However, the in-depth genetic information and structural motifs of *T. nicobarica* mitogenome is still anonymous to the scientific communities. To fill the gap of knowledge, the present study aimed to determine the complete mitogenome of *T. nicobarica* from AN archipelago, India. The comparative analyses were confronted to check the structure and variation within the Tupaiidae mitogenomes. The phylogenetic analyses and divergence time were estimated to infer the evolutionary relationship of *T. nicobarica* comparing with other Tupaiidae species. Further, an additional dataset of mitochondrial *16S rRNA* was also constructed to clarify the taxonomic rank of the extant subspecies of *T. nicobarica* in the Great and Little Nicobar Island.

## Materials and methods

### Sample collection, mitochondria separation, and DNA extraction

The museum sample of *T. nicobarica* is vouchered in 70% ethanol at the National Zoological Collections of Andaman and Nicobar Regional Centre, Zoological Survey of India, which was collected from the Galathia, Campbell bay (06.83 N 93.87 E) on 30th November 2018 (contact person: Govindarasu Gokulakrishnan, Email: gokul7701@gmail.com). No treeshrew was killed as the collected specimen was a natural kill; hence no prior permission was required in the present study. Before preserving, the muscle tissue sample was aseptically collected from the hind leg of the specimen with ample attention and stored in 70% ethanol at Mammal and Osteology section, Zoological Survey of India, Kolkata under voucher ID (Reg. No. 28532) for downstream molecular investigation. The collection locality map was prepared by the online platform (https://noaa.maps.arcgis.com). We used whole mitochondria for the extraction of mitochondrial DNA as per standard protocol^29^. The tissue sample was homogenized with 1 ml buffer containing 0.32 M Sucrose, 1 mM EDTA, and 10 mM TrisHCl by the WiseTis HG-15 homogenizer. To remove the nuclei and cell debris, the working mixture was centrifuged at 700 g for 5 min at 4 °C. The supernatant was collected in 1.5 ml centrifuge tube and centrifuged at 12,000 g for 10 min at 4 °C to precipitate the mitochondrial pellet. The pellet was re-suspended in 500 µl of buffer (50 mM TrisHCl, 25 mM of EDTA, 150 mM NaCl) and incubated at 56 °C for 1–2 h along with 20 µl of proteinase K (20 mg/ml). The mitochondrial DNA was extracted by using QIAamp DNA Investigator Kit (QIAGEN Inc.) and the eluted volume was reduced to 100 µl to increase the mtDNA concentration. The quality of the extracted mtDNA was checked through 1% agarose gel electrophoresis, and the concentration was quantified with a NANODROP 2000 spectrophotometer (Thermo Scientific).

### Sequencing, assembly and annotation

The complete mitogenome sequence and assembly were carried out at PHIXGEN Pvt. Ltd. Gurugram, India (http://www.phixgen.com). The mitochondrial DNA (> 100 ng) was used in Illumina TruSeq Nano DNA HT library preparation kit for library assembly (Illumina, Inc, USA). The mtDNA was fragmented by ultra-sonication (Covaris M220, Covaris Inc., Woburn, MA, USA) and the A-tailed fragments were joined with the sequencing indexed adapters done by the Illumina kit. The mtDNA fragments (450 bp) were selected through sample purification. The amplified PCR library was examined using Bioanalyzer 2100 (Agilent Technologies, Inc., Waldbronn, Germany) with high sensitivity DNA chips. Total > 4 million raw reads were generated through Illumina NextSeq500 (150 × 2 chemistry) (Illumina, Inc, USA). The high-quality reads were downsampled to 2 million using Seqtk (https://github.com/lh3/seqtk) and iterative assembly was performed by using NOVOPlasty v2.6.7 using default parameters^30^. The mitogenome of *T. belangeri* (accession no. NC_002521) was used as a reference seed to start the assembly. The typical circular representation of the generated mitogenome of *T. nicobarica* was plotted by CGView Server (http://stothard.afns.ualberta.ca/cgview_server/) with default parameters^31^. Further, the contig was subjected to confirmation by the MITOS v806 online webserver (http://mitos.bioinf.uni-leipzig.de). The direction and arrangements of PCGs, tRNAs, and rRNAs were confirmed through MITOS online server (http://mitos.bioinf.uni-leipzig.de)^32^. The start and stop codons of each PCG were assured through the Open Reading Frame Finder web tool (https://www.ncbi.nlm.nih.gov/orffinder/) on the basis of vertebrate mitochondrial genetic code and other publicly available reference sequences of Tupaiidae. The mitogenome was submitted to the GenBank database (Accession No. MW751815) using the NCBI Bankit submission tool.

### Dataset construction and comparative analyses

On the basis of taxonomic classification, the mitogenomes of five Tupaiidae species were downloaded from GenBank and merged in the dataset for comparative analysis (Supplementary Table S1). The genome sizes and nucleotide compositions of all the studied species were calculated using MEGA X^33^. To calculate the base composition skew, we utilized previously known formula: AT skew = (A − T)/(A + T), GC skew = (G − C)/(G + C)^34^. The overlapping regions and intergenic spacers of *T. nicobarica* and other Tupaiidae species mitogenomes were calculated manually. The pairwise test of the synonymous (Ks) and non-synonymous (Ka) substitutions were calculated between *T. nicobarica* and other Tupaiidae species using DnaSPv6.0^35^. The comparative analysis of Relative Synonymous Codon Usage (RSCU) and relative abundance of amino acids were also calculated using MEGA X. The secondary structures of tRNA genes were affirmed by tRNAscan-SE Search Server 2.0 (http://lowelab.ucsc.edu/tRNAscan-SE/) and ARWEN 1.2^36,37^. To speculate the putative domains and motif, the CR of *T. nicobarica* and other Tupaiidae species was screened from the database. The tandem repeats within the CR were predicted by the online Tandem Repeats Finder web tool (https://tandem.bu.edu/trf/trf.html)^38^.

### Phylogenetic analysis and divergence time estimation

To assess the phylogenetic relationship, the dataset was constructed with the representatives of Scandentia, Dermoptera, Primates, Lagomorphs, and Rodents based on the previous literatures^15,25,39,40^. The 13 PCGs of 14 mitogenomes were aligned and concatenated using TranslatorX (with MAFFT algorithm with L-INS-i strategy and GBlocks parameters) and SequenceMatrix v1.7.84537^41,42^. The best fit model (GTR + I + G) was estimated by PartitionFinder 2 using lowest Bayesian information criterion (BIC) criterion^43^ and the maximum-likelihood (ML) tree was constructed using the IQ-Tree web server with 1000 bootstrap support^44^. The estimation of divergence times among Tupaiidae species were calculated by Bayesian relaxed clock method in BEAST v2.4.7^45^. The GTR + I + G substitution model, empirical base frequencies, and relaxed uncorrelated log-normal clock with the Yule speciation model was applied as Tree prior. A total of four fossil calibration points were applied in the phylogeny to constraint the analysis as described in the previous study^15,46–48^: (1) node A, 18 million years ago (MYA) log-normal prior as the minimum age of *Tupaia* based on the fossil of *T. miocenica*, (2) node B, 23 MYA log-normal prior as the minimum age for the split between *Pygathrix* and *Hylobates*, (3) node C, a normal prior for the primate outgroups with a mean of 77.5 MYA, and (4) node D, the total tree height is considered as a normal prior with a mean of 90 MYA for Scandentia, Dermoptera, and Primates. Two independent Markov chain Monte Carlo (MCMC) runs were performed for 1,000,000 generations with 25% burn-in and trees sampled every 2000 generations. To combine these runs, log files were imported into LogCombiner of the BEAST package. The adequate MCMC mixing and convergence were estimated using the effective sample size (ESS) values (> 200 in all parameters) in Tracer v.1.7.1^49^. To reconstruct the Bayesian phylogeny, TreeAnnotator was used which is included in the BEAST package. The consensus tree was further visualized by FigTree 1.4.4 with 95% higher probability density (HPD) values on divergence time^50^.

### Analyses of *16S rRNA* dataset

To check the taxonomic rank of two named subspecies, *T. nicobarica nicobarica* from the Great Nicobar Island and *T. nicobarica surda* from the Little Nicobar Island, an additional dataset of *16S rRNA* gene was constructed, including 18 database sequences of 12 Tupaiidae species known from South and Southeast Asian countries (Supplementary Table S2). The *16S rRNA* sequence of the Sunda flying lemur, *Galeopterus variegatus* (Accession No. NC_004031) was used as an out-group in the second dataset. The genetic distances were calculated using MEGA X and the best fit model for this dataset was estimated using Mr. MODELTEST v2with lowest BIC (Bayesian Information Criterion) score^51^. The Bayesian tree was constructed in Mr. Bayes 3.1.2 by selecting nst = 6 for GTR + G + I model and four (one cold and three hot) metropolis-coupled Markov Chain Monte Carlo (MCMC), was run for 10,000,000 generations with 25% burn in with trees saving at every 100 generations^52^. The MCMC analysis was used to generate the convergence metrics, till the standard deviation (SD) of split frequencies reached under 0.01 and the potential scale reduction factor (PSRF) for all parameters approached 1.0. To represent the generated BA tree, the web based iTOL tool (https://itol.embl.de/) was used^53^.

## Results and discussion

### Mitogenome structure and organization

**Figure 1:**
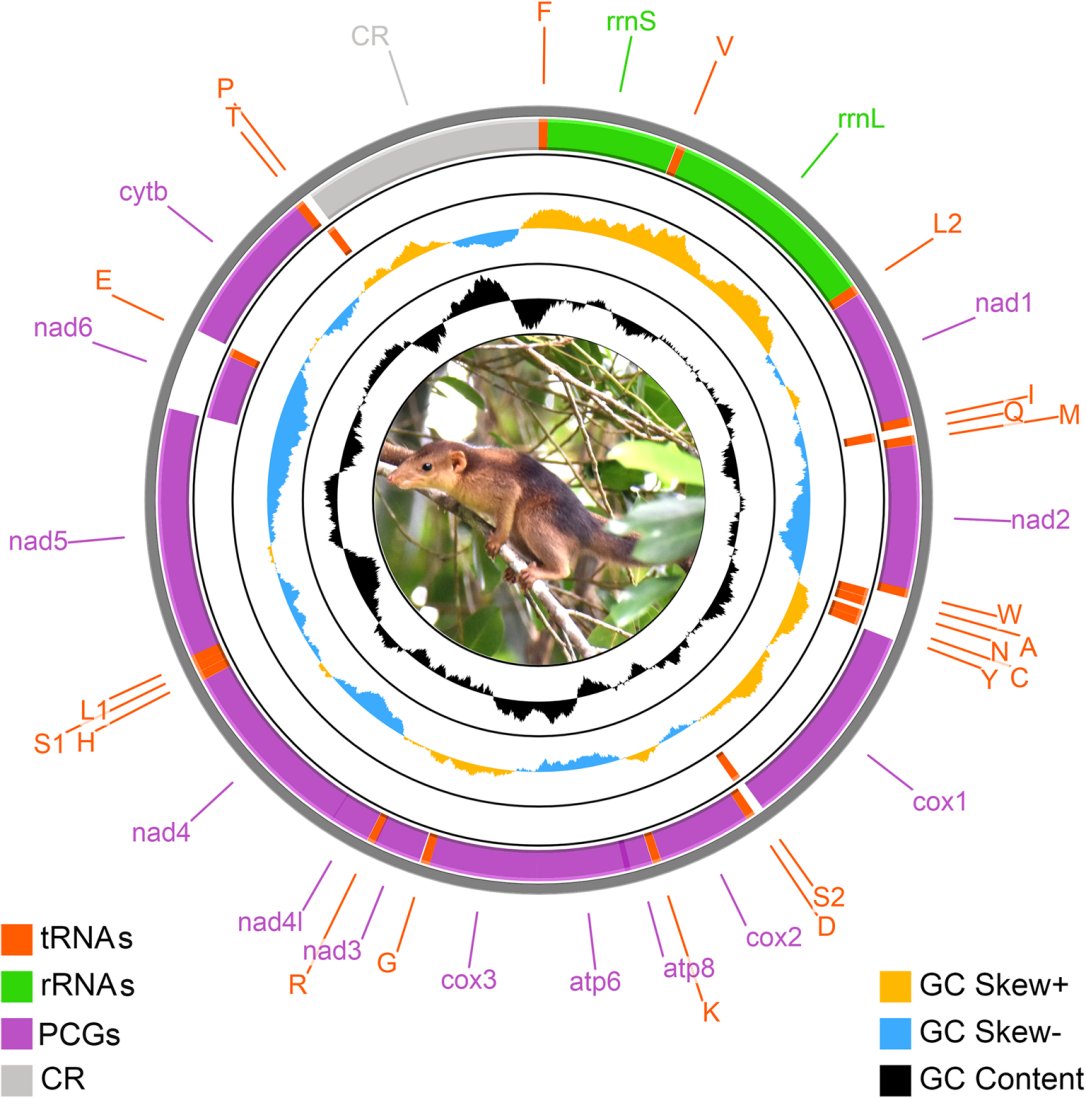
The species photograph and mitochondrial genome of *T. nicobarica*. Protein-coding genes are marked by orcid color boxes (first ring from the outside refers genes in positive strand, while the second ring from the outside refers genes in negative strand), rRNA genes are marked by green color boxes, tRNA genes are marked by red color boxes, and control region is marked by grey color box. tRNAs are encoded according to their single-letter abbreviations. The GC content is plotted using a black sliding window; GC-skew is plotted using orange and blue color sliding windows as deviation from the average of the complete mitogenome. The figure was illustrated using CGView online server (http://cgview.ca/) with default parameters. The species photograph taken by Govindarasu Gokulakrishnan and circular map was merged manually in Adobe Photoshop CS 8.0.

The mitogenome (17,164 bp) of the endangered Nicobar treeshrew, *T. nicobarica* was determined in the present study (GenBank accession no. MW751815). The mitogenome contained 37 genes, comprising 13 PCGs, 22 tRNAs, 2 rRNAs, and a major non-coding CR. Among them, nine genes (*nad6* and eight tRNAs) were placed on the negative strand, while the remaining 28 genes were placed on the positive strand (Table 1, Fig. 1). In the order Scandentia, the length of the Tupaiidae mitogenome varied from 16,183 bp (*T. montana*) to 17,164 bp (*T. nicobarica*). All Tupaiidae species showed the same gene arrangement as observed in typical vertebrate’s mitogenome^54^. The nucleotide composition of the *T. nicobarica* mitogenome was A + T biased (58.3%), as in all Tupaiidae species ranging from 58.3% (*T. nicobarica*) to 59.72% (*T. tana*) (Table 2). The AT skew and GC skew were 0.11 and − 0.30 in the mitogenome of *T. nicobarica*. The comparative analysis showed that the AT skew ranged from 0.08 to 0.11 and the GC skew from − 0.28 to − 0.30 (Table 2). A total of 14 overlapping regions with a total length of 87 bp were identified in *T. nicobarica* mitogenome. The longest overlapping region (43 bp) was observed between the ATP synthase F0 subunit 8 (*atp8*) and ATP synthase F0 subunit 6 (*atp6*). Further, a total of 14 intergenic spacer regions with a total length of 68 bp were observed in *T. nicobarica* mitogenome with the longest region (33 bp) between tRNA-Asparagine (*trnN*) and tRNA-Cysteine (*trnC*) (Supplementary Table S3).

**Table 1:** List of annotated mitochondrial genes of the Nicobar treeshrew *T. nicobarica*.

Gene	Direction	Location	Size (bp)	Anti- codon	Start codon	Stop codon	Intergenic Nucleotides
*trnF*	+	1–66	66	GAA	−	−	− 1
*rrnS*	+	66–1013	948	−	−	−	4
*trnV*	+	1018–1084	67	TAC	−	−	0
*rrnL*	+	1085–2655	1571	−	−	−	0
*trnL2*	+	2656–2730	75	TAA	−	−	2
*nad1*	+	2733–3689	957	−	ATG	TAG	− 2
*trnI*	+	3688–3756	69	GAT	−	−	− 3
*trnQ*	−	3754–3824	71	TTG	−	−	− 1
*trnM*	+	3824–3892	69	CAT	−	−	0
*nad2*	+	3893–4936	1044	−	ATC	TAG	− 2
*trnW*	+	4935–5001	67	TCA	−	−	4
*trnA*	−	5006–5075	70	TGC	−	−	1
*trnN*	−	5077–5149	73	GTT	−	−	33
*trnC*	−	5183–5249	67	GCA	−	−	0
*trnY*	−	5250–5315	66	GTA	−	−	1
*cox1*	+	5317–6864	1548	−	ATG	AGA	− 5
*trnS2*	−	6860–6928	69	TGA	−	−	4
*trnD*	+	6933–7001	69	GTC	−	−	0
*cox2*	+	7002–7685	684	−	ATG	TAG	1
*trnK*	+	7687–7750	64	TTT	−	−	2
*atp8*	+	7753–7956	204	−	ATG	TAA	− 43
*atp6*	+	7914–8594	681	−	ATG	TAA	− 1
*cox3*	+	8594–9379	786	−	ATG	TAA	− 1
*trnG*	+	9379–9445	67	TCC	−	−	9
*nad3*	+	9455–9802	348	−	ATT	TAG	− 10
*trnR*	+	9793–9858	66	TCG	−	−	1
*nad4l*	+	9860–10,156	297	−	ATG	TAA	− 7
*nad4*	+	10,150–11,527	1378	−	ATG	T(AA)	0
*trnH*	+	11,528–11,596	69	GTG	−	−	0
*trnS1*	+	11,597–11,655	59	GCT	−	−	−1
*trnL1*	+	11,655–11,724	70	TAG	−	−	−9
*nad5*	+	11,716–13,536	1821	−	ATA	TAG	2
*nad6*	−	13,539–14,060	522	−	ATG	AGG	0
*trnE*	−	14,061–14,128	68	TTC	−	−	3
*cob*	+	14,132–15,271	1140	−	ATG	TAG	− 1
*trnT*	+	15,271–15,338	68	TGT	−	−	1
*trnP*	−	15,340–15,407	68	TGG	−	−	0
*CR*		15,408–17,164	1757	−	−	−	−

**Table 2:** Nucleotide composition of the mitochondrial genomes of different treeshrew mtDNA.

Species name	Size (bp)	A%	T%	G%	C%	G + C%	AT‐skew	GC‐skew
**Complete mitogenome**								
*T. nicobarica*	17,164	32.56	25.74	14.54	27.13	41.70	0.11	− 0.30
*T. belangeri*	16,754	32.70	26.55	14.37	26.38	40.75	0.10	− 0.29
*T. minor*	16,187	32.06	26.83	14.77	26.32	41.09	0.08	− 0.28
*T. montana*	16,183	32.07	26.63	14.79	26.52	41.30	0.09	− 0.28
*T. splendidula*	16,189	32.33	26.72	14.54	26.17	40.71	0.09	− 0.28
*T. tana*	16,186	32.52	27.20	14.38	25.89	40.28	0.08	− 0.28
**Protein-coding genes (PCGs)**								
*T. nicobarica*	11,410	31.60	26.32	13.31	28.74	42.05	0.09	− 0.36
*T. belangeri*	11,394	30.50	28.30	14.30	26.91	41.21	0.04	− 0.31
*T. minor*	11,387	30.24	28.45	14.49	26.82	41.31	0.03	− 0.30
*T. montana*	11,389	30.25	28.22	14.49	27.04	41.53	0.03	− 0.30
*T. splendidula*	11,362	30.50	28.37	14.33	26.81	41.14	0.04	− 0.30
*T. tana*	11,389	30.89	28.72	14.00	26.39	40.39	0.04	− 0.31
**tRNA genes (tRNAs)**								
*T. nicobarica*	1497	34.06	26.78	17.03	22.11	39.14	0.11	− 0.12
*T. belangeri*	1564	31.91	29.35	20.33	18.41	38.75	0.04	0.05
*T. minor*	1493	30.94	30.01	20.76	18.29	39.05	0.02	0.06
*T. montana*	1496	31.15	29.95	20.52	18.38	38.90	0.02	0.05
*T. splendidula*	1496	31.75	30.21	20.12	17.91	38.03	0.02	0.06
*T. tana*	1497	32.00	29.86	19.97	18.17	38.14	0.03	0.05
**rRNA genes (rRNAs)**								
*T. nicobarica*	2519	35.84	22.70	18.69	22.74	41.44	0.22	− 0.09
*T. belangeri*	2520	36.11	22.86	18.73	22.30	41.03	0.22	− 0.09
*T. minor*	2516	35.41	23.21	18.64	22.73	41.38	0.21	− 0.10
*T. montana*	2508	35.33	23.01	18.74	22.93	41.67	0.21	− 0.10
*T. splendidula*	2514	35.52	23.31	18.70	22.47	41.17	0.21	− 0.09
*T. tana*	2510	35.54	23.86	18.53	22.07	40.60	0.20	− 0.09
**Control regions (CRs)**								
*T. nicobarica*	1757	33.23	25.72	14.34	26.63	40.97	0.12	− 0.30
*T. belangeri*	1350	34.37	28.37	13.11	24.15	37.26	0.10	− 0.30
*T. minor*	788	30.20	29.95	16.50	23.35	39.85	0.00	− 0.17
*T. montana*	790	30.51	29.11	16.46	23.92	40.38	0.02	− 0.18
*T. splendidula*	778	31.75	29.18	16.32	22.75	39.07	0.04	− 0.16
*T. tana*	789	30.42	28.90	16.48	24.21	40.68	0.03	− 0.19

The A + T biases of the complete mitogenome, PCGs, tRNAs, rRNA, and CRs were calculated by AT‐skew = (A − T)/(A + T) and GC‐skew = (G − C)/(G + C), respectively.

### Protein-coding genes

The total length of PCGs was 11,410 bp in *T. nicobarica*, which represents 66.47% of the complete mitogenome. The nucleotide composition of the *T. nicobarica* PCGs was A + T biased (57.95%), as in all Tupaiidae species ranging from 57.95% (*T. nicobarica*) to 59.61% (*T. tana*) (Table 2). The AT skew and GC skew were 0.09 and − 0.36 in the PCGs of *T. nicobarica* (Table 2). Most of the PCGs of *T. nicobarica* initiated with an ATG start codon; however, the ATC initiation codon was found in the NADH dehydrogenase subunit 2 (*nad2*), ATT in NADH dehydrogenase subunit 3 (*nad3*), ATA in NADH dehydrogenase subunit 5 (*nad5*). The TAG termination codon was used by six PCGs, TAA by four PCGs, AGA by Cytochrome oxidase subunit 1 (*cox1*), AGG by NADH dehydrogenase subunit 6 (*nad6*), and incomplete T(AA) by NADH dehydrogenase subunit 4 (*nad4*), respectively. The comparative study revealed that, most of the PCGs in other Tupaiidae species were initiated by ATG start codon and terminated by TAA stop codon (Supplementary Table S4).

The analysis of mitogenome for detecting positive selection of PCGs assists to understand the influences of natural selection in evolution and protein function^55,56^. The comparison of synonymous (Ks) and nonsynonymous (Ka) substitution rates in PCGs, witnessed for Darwinian selection and adaptive molecular evolution^57,58^. It is reported that, for positive selection Ka/Ks > 1, for neutrality Ka/Ks = 1, and for negative selection Ka/Ks < 1^59^. This approach has the benefit to reveal the natural selection acting on PCGs. Thus, to investigate the evolutionary rates between homologous gene pairs, Ka/Ks substitutions were calculated and compared with six Tupaiidae species. The average Ka/Ks values of 13 PCGs varied from 0.006 (*cox1*) to 0.153 (*atp8*) and resulted in the following order: *cox1* < *cox3* < *cox2* < *atp6* < *nad3* < *cytb* < *nad1* < *nad4* < *nad6* < *nad4l* < *nad5* < *nad2* < *atp8* (Supplementary Table S5, Supplementary Fig. S1). Most of the PCGs show Ka/Ks values of < 1, which indicated a strong negative selection among the studied Tupaiidae species, that reflects natural selection works against deleterious mutations with negative selective coefficients as highlighted general patterns in other vertebrates^60^. The comparative RSCU analysis indicated a significant fall in the frequency of GCG codon in Alanine (Ala) was observed in *T. nicobarica*, *T. montana*, *T. minor*, *T. splendidula*, and *T. tana*, except in *T. belangeri* with CCG in Proline (Pro) (Supplementary Fig. S2).

### Ribosomal RNA and transfer RNA genes

The total length of two rRNA genes of *T. nicobarica* was 2,519 bp, compared to a range from 2,508 bp (*T. montana*) to 2,520 bp (*T. belangeri*) among other Tupaiidae species in the present dataset. The AT content within rRNA genes was 58.56%, while the AT and GC skew were 0.22 and − 0.09 respectively observed in *T. nicobarica* rRNAs (Table 2). A total of 22 tRNAs were found in the *T. nicobarica* mitogenome with a total length of 1,497 bp. In other Tupaiidae species, the length of tRNAs varied from 1,493 bp (*T. minor*) to 1,564 bp (*T. belangeri*). The AT content within tRNA genes was 60.86%, while the AT and GC skew were 0.11 and − 0.12, respectively observed in *T. nicobarica* tRNAs (Table 2). Most of the tRNA genes were predicted to be folded into classical cloverleaf structures, except *trnS1* (without DHU stem and loop) and *trnK* (without DHU loop) (Supplementary Fig. S3). The conventional pairings (A=T and G≡C) were observed in most of the tRNAs bases^61^; however, wobble base pairing was observed in the stem of 14 tRNAs (*trnA*, *trnN*, *trnQ*, *trnE*, *trnC*, *trnG*, *trnL1*, *trnK*, *trnL2*, *trnP*, *trnS2*, *trnT*, *trnY*, and *trnW*) (Supplementary Fig. S3). The wobble base pairing is a key feature of RNA structure and often substitutes the conventional base pairs due to thermodynamic stability. These characteristics play crucial functional roles in a wide range of phenomena^62^. Thus, the comparisons of tRNAs secondary structures are crucial for inferring the structural and functional features of the mitogenomes^63^.

### Control regions

**Figure 2:**
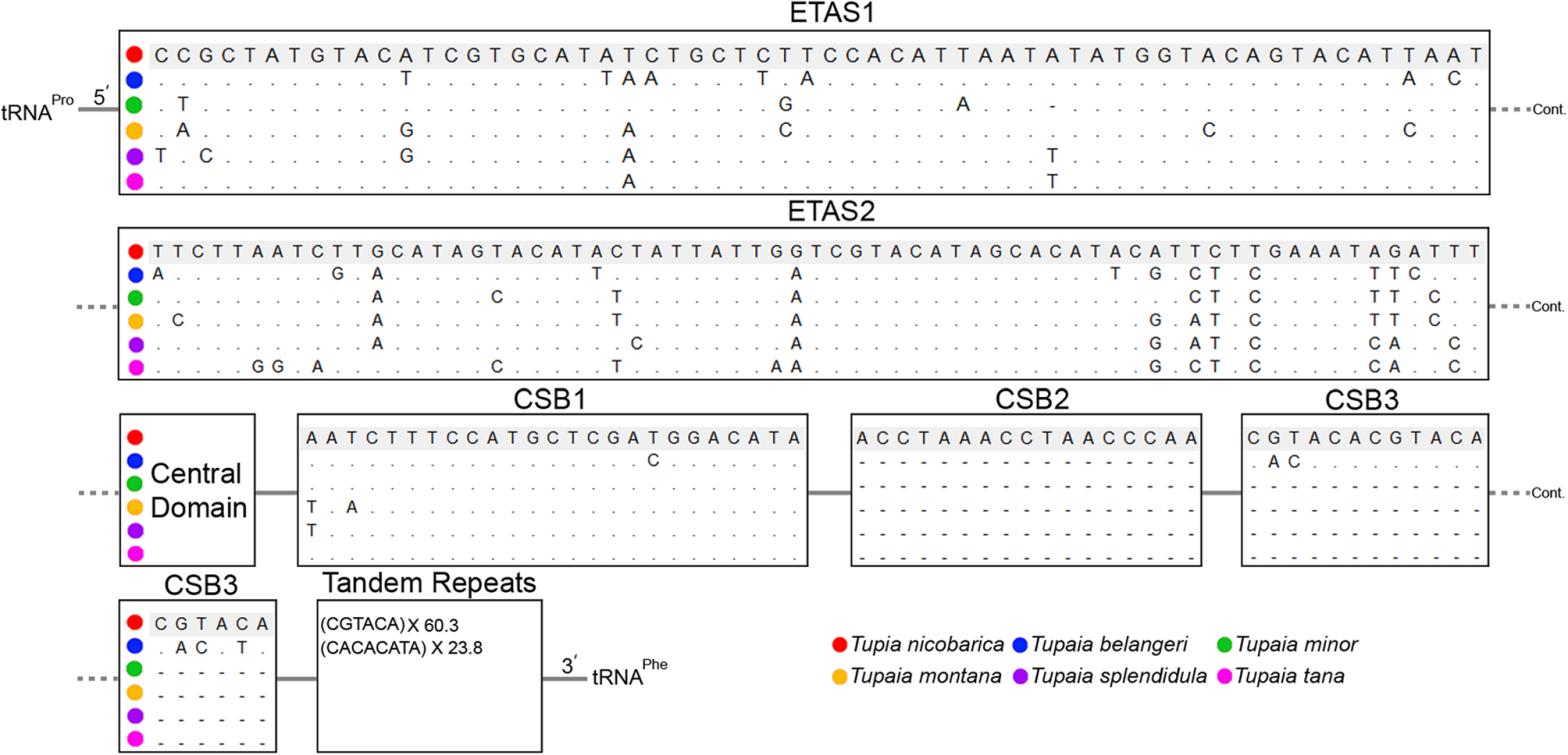
Comparison of nucleotide composition in different domains of control regions (CRs) and tandem repeats of six Tupaiidae species. The nucleotide compositions were compared through MEGAX software and the tandem repeats were predicted by the online Tandem Repeats Finder web tool (https://tandem.bu.edu/trf/trf.html). The figure was edited manually in Adobe Photoshop CS 8.0.

The CR of *T. nicobarica* was typically distributed with three functional domains: extended termination associated sequences (ETAS), central domain (CD), and the conserved sequence block (CSB), as observed in other mammalian mitochondrial CRs^25,64^. Although, the ETAS and CSB domains contain varying numbers of tandem repeats, the CD domain consists with highly conserved sequences. Hence, the pattern of CR was varied among different mammals, including Tupaiidae (Scandentia). The total length of *T. nicobarica* CR was 1,757 bp, compared to a range of 778 bp (*T. splendidula*) to 1,757 bp (*T. nicobarica*) in the present dataset. In the *T. nicobarica* CR, the AT and GC skew was 0.12 and − 0.30 (Table 2). The CR is also involved in the initiation of replication and is positioned between *trnP* and *trnF* for most of the Tupaiidae including *T. nicobarica*. The ETAS domain was divided into two regions: ETAS1 (60 bp) and ETAS2 (67 bp), while the CSB domain was further divided into three regions: CSB1 (25 bp), CSB2 (17 bp), and CSB3 (18 bp). After CSB3, a six base pair (CGTACA) tandem repeats were found 60.3 times in *T. nicobarica*, while eight base pair (CACACATA) were found 23.8 times in *T. belangeri* (Fig. 2). Due to the short nucleotide length, no tandem repeats were found in other Tupaiidae species CRs. The structural features of CR play an important function in influencing transcription and replication in the mitochondrial genome^65,66^. The present study evaluated the genetic features of CR among the studied Tupaiidae species mitogenomes including *T. nicobarica* that will be helpful to speculate the evolutionary pattern of this group.

### Phylogenetic inference

**Figure 3:**
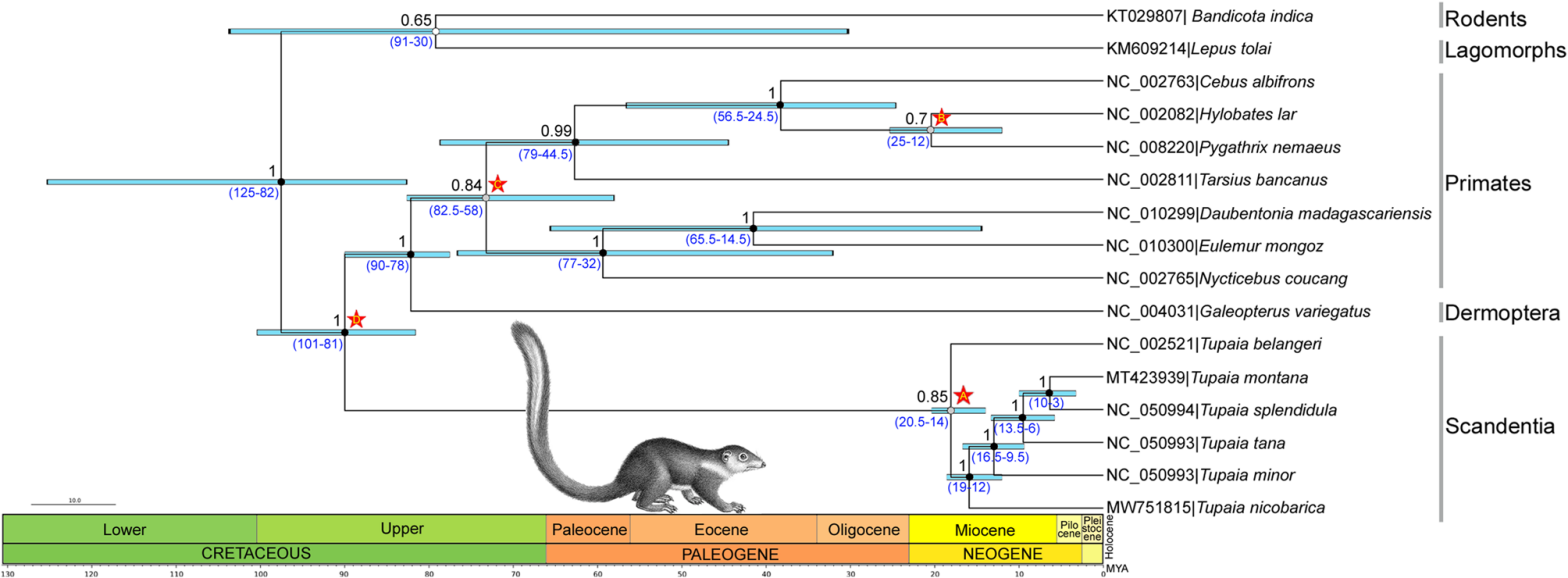
Bayesian inference showed the molecular timescale for Tupaiidae species evolution compared with other Primates and Dermoptera species as well as Lagomorphs and Rodents as out-group. Posterior probabilities were represented by black digit along with each node. The divergence times (in MYA) were estimated by four calibration points (marked by red stars) with GTR + I + G substitution model and relaxed uncorrelated log-normal clock with the Yule speciation model in BEAST v2.4.7. Blue bars represent 95% highest probability density (HPD) around mean estimates of divergence times. The range of the estimated divergence times were marked by values in blue along with each node. Treeshrew artwork was acquired from web (https://www.wpclipart.com; Paul Sherman) and edited manually in Adobe Photoshop CS 8.0.

The phylogenetic position of Scandentia is repetitively argued and examined within the eutherian tree^15,25,39,40^. The treeshrews are widely considered as living fossils due to their approximating ancestral lineages with primates^67^. Based on the anatomical evidence, Primates, Chiroptera, Dermoptera, and Scandentia were hypothetically within the superordinal clade Archonta without considering the paleontological or molecular evidence^68,69^. Later on, the phylogenetic position of Scandentia has been studied based on the complete mitochondrial DNA sequences of wider group of taxa and corroborated a closer relationship with Lagomorpha^24,25,39^. Further, multiple loci of mitochondrial genes has been assessed to check the phylogeny of treeshrews and diversification and the timescale of diversification in Southeast Asia^15,27,40,70^. The present ML and BA phylogenies clearly discriminate *T. nicobarica* from other congeners and are congruent with earlier evolutionary hypotheses of Scandentia (Fig. 3, Supplementary Fig. S4). Further, using four calibration points from earlier studies, the present mitogenome-based dating analysis indicates that, the Tupaiidae species (Scandentia) were diverged from Primates and Dermoptera during the Cretaceous period (81–101 MYA). However, the basal node of Scandentia, Primates and Dermoptera was diverged from the Lagomorphs and Rodents during the same Cretaceous period (82–125 MYA). As a whole the divergence time estimations are little deviated due to the exclusion of Lagomorphs and Rodents in earlier analysis^15^. However, the representative of Scandentia family members (Ptilocercidae and Tupaiidae) in earlier analysis revealed that, they were diverged during Neogene to Paleogene period. Due to the lack of mitogenomic information of all extant Tupaiidae species, we restricted our analysis with few representative species. Diversification of the studied Tupaiidae species occurred during the Pliocene to Miocene epoch (3–20.5 MYA). The endemic Nicobar treeshrew, *T. nicobarica* was diverged from the common ancestor lineages of other Tupaiidae species during the Miocene epoch (12–19 MYA) (Fig. 3).

**Figure 4:**
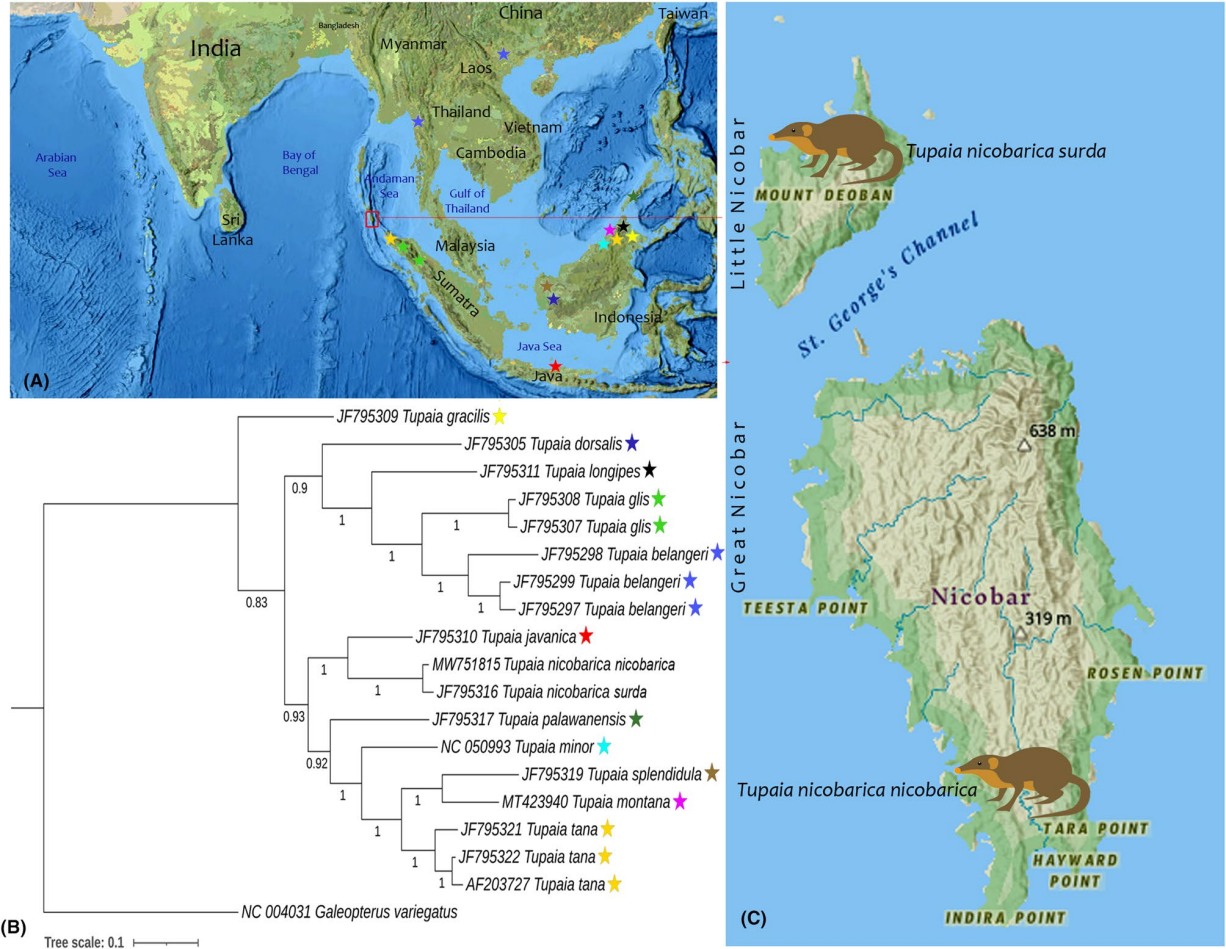
Genetic status of two known subspecies of *T. nicobarica* based on *16S rRNA* sequences. (**A**) Map showing the distribution of other comparative Tupaiidae species in the present phylogeny. The first author (S.K.) prepared the map by using software QGIS 2.6.1 (http://www.qgis.org), the artwork of *T. nicobarica* subspecies and edited manually in Adobe Photoshop CS 8.0. (**B**) BA Phylogeny showed distinct clustering of *T. nicobarica* subspecies and other Tupaiidae species. Numbers on the nodes are posterior probabilities. (**C**) Distribution pattern of *T. nicobarica nicobarica* and *T. nicobarica surda* in the Great and Little Nicobar Island, respectively.

Further, based on *16S rRNA* genes (1667 bp), we evaluated the status of two known subspecies of *T. nicobarica* from the Great and Little Nicobar Islands. The *T. nicobarica nicobarica* and *T. nicobarica surda* showed cohesive clustering in the BA tree as compared with other species (Fig. 4). Both the subspecies depicted 11 variable sites and maintained less genetic distance (0.7%) with each other. The *16S rRNA* based topology showed a sister relationship of *T. nicobarica* with *T. javanica*, distributed in Sumatra and Java.

### Biogeographic connection and conservation implication

The tectonic drifts allowed multiple possibilities for dispersal and colonization events of many animals into the same or distant geographical distribution. Due to the adjacent biogeographic realms, the biological affinities between the Indian mainland and Southeast Asia have been well documented^71^. However, the faunal diversity of the AN archipelago and their biotic networks is still anonymous in spectacular aspects. The bathymetric study evidenced that the well-developed seamounts have been detected on the Andaman seafloor, which extended up to Sumatra and Java Islands^72,73^. Considering the skeletal variation, the treeshrew species showed intraspecific variation depending upon their distribution in mainland and island ecosystems^74^. A recent molecular study also elucidates the biogeographic connection of smaller mammals in the AN archipelago with the Indo-Malayan and Sundaic realms^7^. The present mitogenome based phylogeny also manifested the close relationship of *T. nicobarica* with *T. minor*, *T. tana*, *T. splendidula*, and *T. montana* (distributed in Thailand, Peninsular and East Malaysia, Brunei Darussalam, Sumatra, and Indonesia) as compared with the widespread species *T. belangeri* known from South and Southeast Asia. Further, the single gene (16S rRNA) based phylogeny showed sister relationship of *T. nicobarica* with *T. javanica* (distributed in Sumatra and Java) as compared with other congeners.

The two subspecies of *T. nicobarica* were discriminated only by their different distributional pattern in two distinct islands. Prior to this study, they were neither examined morphologically nor tested genetically to assure their taxonomic status. The present molecular based assessment clearly distinguished two subspecies with 0.7% genetic distance by 16S rRNA gene. This preliminary molecular information will help for rapid and reliable identification of this highly threatened and endemic species from the Great and Little Nicobar Islands. However, further research can be done with the extensive sampling and generation of microsattelite data to substantiate their population genetic structure to formulate precise conservation action plans.

Considering the conservation implication, the previous studies reported that, this arboreal mammal species confronted several threats due to the forest loss and fragmentation, and ongoing road construction from Galathia to Indira Point at the Great Nicobar Island^75^. Although the species is listed on the IUCN with decreasing population trend, it has not yet listed in the Indian wildlife (Protection) Act, 1972. Other than a single ecology and behavior study and a nest record, no ample assessment has been approached so far^3,76^.

Besides, the treeshrews species were considered as a significant model for studying hepatitis and influenza H1N1 viral infections^19,20^. A recent study characterized the genome sequence to demonstrate the genetic basis of signaling pathways in nervous and immune systems of the Chinese treeshrew (*Tupaia belangeri chinensis*) and evidenced as a potential model for biomedical research^21^. The *T. belangeri chinensis* also maintained sufficient intraspecific variation (5.4–9.5%) with the Northern Treeshrew, *T. belangeri* in all 13 PCGs. Hence, the generation of molecular information from different geographical region is crucial for elucidating the actual evolutionary history of this small mammal species.

We propose the whole genome sequencing of *T. nicobarica* is essential as a genetic resource for conservation purposes. The genome sequence will also assist to predict the signaling pathways linked with many pathogenic microorganisms as well as able to develop potential mitigations programs in advance. As the population of this treeshrew is confined to the insular habitats in the Nicobar Islands, we propose an integrated approach with taxonomy, ecology, and further molecular studies to save this endemic species before it reaches to the brink of extinction.

## Supplementary Information

Supplementary Information.

## Data Availability

The following information was supplied regarding the accessibility of DNA sequences: The generated complete mitochondrial genome sequences of *Tupaia nicobarica* are deposited in GenBank of NCBI under accession number MW751815.

## References

[1] Burgin CJ, Colella JP, Kahn PL, Upham NS (2018). How many species of mammals are there?. J. Mamm..

[2] Wilson DE, Mittermeier RA (2018). Handbook of the Mammals of the World, Vol 8: Insectivores, Sloths and Colugos.

[3] Oommen MA, Shanker K (2008). Ecology and behaviuour of endemic treeshrew *Tupaia nicobarica* Zelebor 1869 on Great Nicobar Island, India. J. Bom. Nat. Hist. Soc..

[4] IUCN (2021). The IUCN Red List of Threatened Species, Version 2021–2.

[5] Yahya HSA, Zarri AA (2002). Status, ecology and behaviour of Narcondam Hornbill (*Aceros narcondami*) in Narcondam Island, Andaman and Nicobar Islands, India. J. Bom. Nat. His. Soc..

[6] Datta-Roy A, Karanth KP (2009). The Out-of-India hypothesis: What do molecules suggest?. J. Biosci..

[7] Kamalakannan M, Sivaperuman C, Kundu S (2021). Discovery of a new mammal species (Soricidae: Eulipotyphla) from Narcondam volcanic island, India. Sci. Rep..

[8] Matthews TJ, Rigal F, Triantis KA, Whittaker RJ (2019). A global model of island species–area relationships. Proc. Natl. Acad. Sci. USA.

[9] Upham NS, Esselstyn JA, Jetz W (2019). Ecological causes of uneven diversification and richness in the mammal tree of life. BioRxiv.

[10] Davidar P, Yoganand K, Ganesh T (2001). Distribution of forest birds in the Andaman Islands: Importance of key habitats. J. Biogeogr..

[11] Harikrishnan, S. *et al.* Macroecology of Terrestrial Herpetofauna in Andaman & Nicobar Archipelago. Wildlife Institute of India, Uttarakhand, India. 1–49 (2014).

[12] Kamalakannan M, Venkatraman C (2017). A Checklist of Mammals of India.

[13] Menon V (2014). Indian Mammals—A Field Guide.

[14] Miller GS (1902). Mammals of the Andaman and Nicobar Islands. Proc. U.S Nat Mus..

[15] Roberts TE, Lanier HC, Sargis EJ, Olson LE (2011). Molecular phylogeny of treeshrews (Mammalia: Scandentia) and the timescale of diversification in Southeast Asia. Mol. Phylogenet. Evol..

[16] Oommen MA, Johnsingh AJT, Manjrekar N (2013). Treeshrews. Mammals of South Asia.

[17] Damas J (2020). Broad host range of SARS-CoV-2 predicted by comparative and structural analysis of ACE2 in vertebrates. Proc. Natl. Acad. Sci. USA.

[18] Wardeh M, Baylis M, Blagrove MSC (2021). Predicting mammalian hosts in which novel coronaviruses can be generated. Nat. Commun..

[19] Cao J, Yang E-B, Su J-J, Li Y, Chow P (2003). The tree shrews: Adjuncts and alternatives to primates as models for biomedical research. J. Med. Primatol.

[20] Yang ZF (2013). The tree shrew provides a useful alternative model for the study of influenza H1N1 virus. Virol. J..

[21] Yu F (2013). Genome of the Chinese tree shrew. Nat. Commun..

[22] Pacheco MA (2011). Escalante, Evolution of modern birds revealed by mitogenomics: Timing the radiation and origin of major orders. Mol. Biol. Evol..

[23] Finstermeier K (2013). A mitogenomic phylogeny of living primates. PLoS One.

[24] Arnason U (2020). Mammalian mitogenomic relationships and the root of the eutherian tree. Proc. Natl. Acad. Sci. USA.

[25] Schmitz J, Ohme M, Zischler H (2000). The complete mitochondrial genome of *Tupaia belangeri* and the phylogenetic affiliation of scandentia to other eutherian orders. Mol. Biol. Evol..

[26] Parker D (2020). Little genetic structure in a Bornean endemic small mammal across a steep ecological gradient. Mol. Ecol..

[27] Olson LE, Sargis EJ, Martin RD (2005). Intraordinal phylogenetics of treeshrews (Mammalia: Scandentia) based on evidence from the mitochondrial 12S rRNA gene. Mol. Phylogenet. Evol..

[28] Kundu S (2020). Molecular investigation of non-volant endemic mammals through mitochondrial cytochrome b gene from Andaman and Nicobar archipelago. Mitochondrial DNA B.

[29] Kundu S (2020). The complete mitochondrial genome of the endangered Assam Roofed Turtle, *Pangshura sylhetensis* (Testudines: Geoemydidae): Genomic features and phylogeny. PLoS One.

[30] Dierckxsens N, Mardulyn P, Smits G (2017). NOVOPlasty: De novo assembly of organelle genomes from whole genome data. Nucleic Acids Res..

[31] Grant JR, Stothard P (2008). The CGView Server: A comparative genomics tool for circular genomes. Nucleic Acids Res..

[32] Bernt M (2013). MITOS: Improved de novo metazoan mitochondrial genome annotation. Mol. Phylogenet. Evol..

[33] Kumar S, Stecher G, Li M, Knyaz C, Tamura K (2018). MEGA X: Molecular evolutionary genetics analysis across computing platforms. Mol. Biol. Evol..

[34] Perna NT, Kocher TD (1995). Patterns of nucleotide composition at fourfold degenerate sites of animal mitochondrial genomes. J. Mol. Evol..

[35] Rozas J (2017). DnaSP 6: DNA sequence polymorphism analysis of large data sets. Mol. Biol. Evol..

[36] Laslett D, Canbäck B (2008). ARWEN, a program to detect tRNA genes in metazoan mitochondrial nucleotide sequences. Bioinformatics.

[37] Lowe TM, Chan PP (2016). tRNAscan-SE on-line: Search and contextual analysis of transfer RNAGenes. Nucleic Acids Res..

[38] Benson G (1999). Tandem repeats finder: A program to analyze DNA sequences. Nucleic Acids Res..

[39] Xu L, Chen SY, Nie WH (2012). Evaluating the phylogenetic position of Chinese tree shrew (*Tupaia belangeri chinensis*) based on complete mitochondrial genome: Implication for using tree shrew as an alternative experimental animal to primates in biomedical research. J. Genet. Genom..

[40] Zhou X, Sun F, Xu S, Yang G, Li M (2015). The position of tree shrews in the mammalian tree: Comparing multi-gene analyses with phylogenomic results leaves monophyly of Euarchonta doubtful. Integr. Zool..

[41] Abascal F, Zardoya R, Telford MJ (2010). TranslatorX: Multiple alignment of nucleotide sequences guided by amino acid translations. Nucleic Acids Res..

[42] Vaidya G, Lohman DJ, Meier RJ (2010). SequenceMatrix: Concatenation sofware for the fast assembly of multigene datasets with character set and codon information. Cladistics.

[43] Lanfear R, Frandsen PB, Wright AM, Senfeld T, Calcott B (2016). PartitionFinder 2: New methods for selecting partitioned models of evolution for molecular and morphological phylogenetic analyses. Mol. Biol. Evol..

[44] Trifinopoulos J, Nguyen L-T, von Haeseler A, Minh BQ (2016). W-IQ-TREE: A fast online phylogenetic tool for maximum likelihood analysis. Nucleic Acids Res..

[45] Bouckaert R (2014). BEAST2: A software platform for Bayesian evolutionary analysis. PLoS Comput. Biol..

[46] Mein P, Ginsburg L (1997). Les mammifères du gisement miocène inférieur de Li Mae Long, Thaïlande: Systématique, biostratigraphie et paléoenvironnement. Geodiversitas.

[47] Eizirik E, Murphy WJ, O’Brien SJ, Ross CF, Kay RF (2004). Molecular phylogeny and dating of early primate divergences. Anthropoid Origins. New visions.

[48] Hedges SB, Dudley J, Kumar S (2006). TimeTree: A public knowledge-base of divergence times among organisms. Bioinformatics.

[49] Rambaut A, Drummond AJ, Xie D, Baele G, Suchard MA (2018). Posterior summarisation in Bayesian phylogenetics using Tracer 1.7. Syst. Biol..

[50] Rambaut A (2014). FigTree. Version 1.4.4 Institute of Evolutionary Biology.

[51] Nylander JAA (2004). MrModeltest v2.

[52] Ronquist F, Huelsenbeck JP (2003). MrBayes 3: Bayesian phylogenetic inference under mixed models. Bioinformatics.

[53] Letunic I, Bork P (2007). Interactive Tree Of Life (iTOL): An online tool for phylogenetic tree display and annotation. Bioinformatics.

[54] Anderson S (1982). Complete sequence of bovine mitochondrial DNA conserved features of the mammalian mitochondrial genome. J. Mol. Evol..

[55] Hirsh AE, Fraser HB (2001). Protein dispensability and rate of evolution. Nature.

[56] Bloom JD, Labthavikul ST, Otey CR (2006). Protein stability promotes evolvability. Proc. Natl. Acad. Sci. USA.

[57] Yang Z, Bielawski JP (2000). Statistical methods for detecting molecular adaptation. Trends Ecol. Evol..

[58] Yang ZH, Nielsen R (2000). Estimating synonymous and nonsynonymous substitution rates under realistic evolutionary models. Mol. Biol. Evol..

[59] Nei M, Kumar S (2000). Molecular Evolution and Phylogenetics.

[60] Meiklejohn CD, Montooth KL, Rand DM (2007). Positive and negative selection on the mitochondrial genome. Trends Genet..

[61] Varani G, McClain WH (2000). The G-U wobble base pair: A fundamental building block of RNA structure crucial to RNA function in diverse biological systems. EMBO Rep..

[62] Crick FHC (1966). Codon-anticodon pairing: The wobble hypothesis. J. Mol. Biol..

[63] Takashi PS, Miya M, Mabuchi K (2016). Structure and variation of the mitochondrial genome of fishes. BMC Genom..

[64] Sbisa E, Tanzariello F, Reyes A, Pesole G, Saccone C (1997). Mammalian mitochondrial D-loop region structural analysis: Identification of new conserved sequences and their functional and evolutionary implications. Gene.

[65] Taanman JW (1999). The mitochondrial genome: Structure, transcription, translation and replication. Biochim. Biophys. Acta.

[66] Shao R, Barker SC, Mitani H, Aoki Y, Fukunaga M (2005). Evolution of duplicate control regions in the mitochondrial genomes of Metazoa: A case study with Australasian Ixodes ticks. Mol. Biol. Evol..

[67] Li Q, Ni X (2016). An early Oligocene fossil demonstrates treeshrews are slowly evolving “living fossils”. Sci. Rep..

[68] Novacek MJ (1992). Mammalian phylogeny: Shaking the tree. Nature.

[69] McKenna MC, Bell SK (1997). Classification of Mammals Above the Species Level.

[70] Roberts TE, Sargis EJ, Olson LE (2009). Networks, trees, and treeshrews: Assessing support and identifying conflict with multiple loci and a problematic root. Syst. Biol..

[71] Garg S, Biju SD (2019). New microhylid frog genus from Peninsular India with Southeast Asian affinity suggests multiple Cenozoic biotic exchanges between India and Eurasia. Sci. Rep..

[72] Rodolfo KS (1969). Bathymetry and marine geology of the Andaman basin, and tectonic implications for Southeast Asia. Geol. Soc. Am. Bull..

[73] Tripathi SK (2017). Morphology of submarine volcanic seamounts from inner volcanic arc of Andaman Sea. Indian J. Geosci..

[74] Sargis EJ, Woodman N, Morningstar NC, Bell TN, Olson LE (2017). Skeletal variation and taxonomic boundaries among mainland and island populations of the common treeshrew (Mammalia: Scandentia: Tupaiidae). Biol. J. Linn. Soc..

[75] Molur, S. *et al. Status of Non-volant Small Mammals: Conservation Assessment and Management Plan (CAMP) Workshop Report* 618 (Zoo Outreach Organisation, 2005).

[76] Kamalakannan M, Gokulakrishnan G, Venkatraman C, Sivaperuman C, Chandra K (2021). First record of a Nicobar treeshrew nest in a fallen palm tree. Mammalia.

