# Supplementary material for: Complete mitogenome of the endangered and endemic Nicobar treeshrew (Tupaia nicobarica) and comparison with other Scandentians
Source: Sci Rep. 2022 Jan 18;12:877. doi: 10.1038/s41598-022-04907-7 (PMC8766473; doi:10.1038/s41598-022-04907-7)

**Supporting information**

**Complete mitogenome of the endangered and endemic Nicobar treeshrew (*Tupaia nicobarica*) and comparison with other Scandentians**

Shantanu Kundu^1^, Avas Pakrashi^1^, Manokaran Kamalakannan^2^, Devkant Singha, Kaomud Tyagi^1^, Dhriti Banerjee^1,2^, Chinnadurai Venkatraman^2^, Vikas Kumar^1*^

1. Centre for DNA Taxonomy, Molecular Systematics Division, Zoological Survey of India, Kolkata 700053, India.

2. Mammal and Osteology Section, Zoological Survey of India, Kolkata 700053, India.

**Table S1.** List of species and their accession numbers used for comparative and phylogenetic analyses.

| **Sl No.** | **Orders** | **Species** | **Accession Nos.** | **Size (**bp**)** | **References** |
| --- | --- | --- | --- | --- | --- |
| 1 | Scandentia | *Tupia nicobarica* | MW751815 | 17164 | This study |
| 2 | Scandentia | *Tupaia belangeri* | NC_002521 | 16754 | Schmitz et al. 2000 |
| 3 | Scandentia | *Tupaia minor* | NC_050993 | 16187 | Parker et al. 2020 |
| 4 | Scandentia | *Tupaia montana* | MT423939 | 16183 | Parker et al. 2020 |
| 5 | Scandentia | *Tupaia splendidula* | NC_050994 | 16189 | Parker et al. 2020 |
| 6 | Scandentia | *Tupaia tana* | NC_050992 | 16186 | Parker et al. 2020 |
| 7 | Dermoptera | *Galeopterus variegatus* | NC_004031 | 16748 | Arnason et al. 2002 |
| 8 | Primates | *Daubentonia madagascariensis* | NC_010299 | 16620 | Arnason et al. 2008 |
| 9 | Primates | *Cebus albifrons* | NC_002763 | 16554 | Arnason et al. 2000 |
| 10 | Primates | *Eulemur mongoz* | NC_010300 | 16686 | Arnason et al. 2008 |
| 11 | Primates | *Hylobates lar* | NC_002082 | 16472 | Arnason et al. 1996 |
| 12 | Primates | *Pygathrix nemaeus* | NC_008220 | 15467 | Sterner et al. 2006 |
| 13 | Primates | *Tarsius bancanus* | NC_002811 | 16927 | Schmitz et al. 2002 |
| 14 | Primates | *Nycticebus coucang* | NC_002765 | 16764 | Arnason et al. 2000 |
| 15 | Lagomorphs | *Lepus tolai* | KM609214 | 17472 | Ding et al. 2016 |
| 16 | Rodents | *Bandicota indica* | KT029807 | 16326 | Wang et al. 2016 |

**Cross References:**

1. Arnason U, Adegoke JA, Bodin K, Born EW, Esa YB, Gullberg A, Nilsson M, Short RV, Xu X, Janke A. 2020. Mammalian mitogenomic relationships and the root of the eutherian tree. Proc Natl Acad Sci USA. 99: 8151-6.
2. Arnason U, Adegoke JA, Gullberg A, Harley EH, Janke A, Kullberg M. 2008. Mitogenomic relationships of placental mammals and molecular estimates of their divergences. Gene. 421: 37-51.
3. Arnason U, Gullberg A, Burguete AS, Janke A. 2000. Molecular estimates of primate divergences and new hypotheses for primate dispersal and the origin of modern humans. Hereditas. 133: 217-28.
4. Arnason U, Gullberg A, [Xu](https://onlinelibrary.wiley.com/action/doSearch?ContribAuthorStored=Xu%2C+Xiufeng) X. 1996. A complete mitochondrial DNA molecule of the white-handed gibbon, *Hylobates lar*, and comparison among individual mitochondrial genes of all hominoid genera. 124: 185-189.
5. Parker LD, Hawkins MTR, Camacho-Sanchez M, Campana MG, West-Roberts JA, Wilbert TR, Lim HC, Rockwood LL, Leonard JA, Maldonado JE. 2020. Little genetic structure in a Bornean endemic small mammal across a steep ecological gradient. Mol Ecol. 29: 4074-4090.
6. Schmitz J, Ohme M, Zischler H. 2000. The complete mitochondrial genome of *Tupaia belangeri* and the phylogenetic affiliation of scandentia to other eutherian orders. Mol Biol Evol. 17: 1334-43.
7. Schmitz J, Ohme M, Zischler H. 2002. The complete mitochondrial sequence of *Tarsius bancanus*: evidence for an extensive nucleotide compositional plasticity of primate mitochondrial DNA. Mol Biol Evol. 19: 544-53.
8. Sterner KN, Raaum RL, Zhang YP, Stewart CB, Disotell TR. 2006. Mitochondrial data support an odd-nosed colobine clade. Mol Phylogenet Evol. 40: 1-7.

Ding L, Chen C, Wang H, Zhang B. Complete mitochondrial DNA sequence of Lepus tolai (Leporidae: Lepus). Mitochondrial DNA A DNA Mapp Seq Anal. 2016 May;27(3):2085-6. doi: 10.3109/19401736.2014.982568. Epub 2014 Nov 12. PMID: 25391036.

Wang S, Cong H, Kong L, Motokawa M, Li Y. Complete mitochondrial genome of the greater bandicoot rat Bandicota indica (Rodentia: Muridae). Mitochondrial DNA A DNA Mapp Seq Anal. 2016 Nov;27(6):4349-4350. doi: 10.3109/19401736.2015.1089495. Epub 2015 Oct 16. PMID: 26471207.

**Table S2.** List of species and their accession numbers used for subspecies level delimitation of *T. nicobarica* based on *16S rRNA*.

| **Sl No.** | **Species** | **Accession Nos.** | **References** |
| --- | --- | --- | --- |
| 1 | *Tupaia nicobarica nicobarica* | MW751815 | This Study |
| 2 | *Tupaia nicobarica surda* | JF795316 | Roberts et al. 2011 |
| 3 | *Tupaia gracilis* | JF795309 |  |
| 4 | *Tupaia dorsalis* | JF795305 |  |
| 5 | *Tupaia longipes* | JF795311 |  |
| 6 | *Tupaia glis* | JF795308 |  |
| 7 | *Tupaia glis* | JF795307 |  |
| 8 | *Tupaia belangeri* | JF795298 |  |
| 9 | *Tupaia belangeri* | JF795299 |  |
| 10 | *Tupaia belangeri* | JF795297 |  |
| 11 | *Tupaia javanica* | JF795310 |  |
| 12 | *Tupaia palawanensis* | JF795317 |  |
| 13 | *Tupaia splendidula* | JF795319 |  |
| 14 | *Tupaia tana* | JF795321 |  |
| 15 | *Tupaia tana* | JF795322 |  |
| 16 | *Tupaia tana* | AF203727 | Springer and Waddell 2000 |
| 17 | *Tupaia minor* | NC_050993 | Parker et al. 2020 |
| 18 | *Tupaia montana* | MT423940 | Parker et al. 2020 |
| 19 | *Galeopterus variegatus* | NC_004031 | Arnason et al. 2002 |

**Cross References:**

1. Parker LD, Hawkins MTR, Camacho-Sanchez M, Campana MG, West-Roberts JA, Wilbert TR, Lim HC, Rockwood LL, Leonard JA, Maldonado JE. 2020. Little genetic structure in a Bornean endemic small mammal across a steep ecological gradient. Mol Ecol. 29: 4074-4090.
2. Roberts TE, Lanier HC, Sargis EJ, Olson LE. 2011. Molecular phylogeny of treeshrews (Mammalia: Scandentia) and the timescale of diversification in Southeast Asia. Mol Phylogenet Evol. 60: 358-72.
3. Arnason U, Adegoke JA, Bodin K, Born EW, Esa YB, Gullberg A, Nilsson M, Short RV, Xu X, Janke A. Mammalian mitogenomic relationships and the root of the eutherian tree. Proc Natl Acad Sci U S A. 2002. 99: 8151-6.

**Table S3.** Comparative analysis of intergenic nucleotides among six Tupaiidae species.

| Gene | ***Tupia nicobarica*** | | | ***Tupaia belangeri*** | | | ***Tupaia minor*** | | | ***Tupaia montana*** | | | ***Tupaia splendidula*** | | | ***Tupaia tana*** | | |
| --- | --- | --- | --- | --- | --- | --- | --- | --- | --- | --- | --- | --- | --- | --- | --- | --- | --- | --- |
|  | Start | Stop | ovl/nc | Start | Stop | ovl/nc | Start | Stop | ovl/nc | Start | Stop | ovl/nc | Start | Stop | ovl/nc | Start | Stop | ovl/nc |
| *trnF* | 1 | 66 | -1 | 1 | 66 | 0 | 1 | 66 | 0 | 1 | 66 | 0 | 1 | 66 | 0 | 1 | 66 | 0 |
| *rrnS* | 66 | 1013 | 4 | 67 | 1014 | 0 | 67 | 1015 | 0 | 67 | 1010 | 0 | 67 | 1010 | 0 | 67 | 1011 | 0 |
| *trnV* | 1018 | 1084 | 0 | 1015 | 1080 | 0 | 1016 | 1082 | 0 | 1011 | 1078 | 0 | 1011 | 1078 | 0 | 1012 | 1079 | 0 |
| *rrnL* | 1085 | 2655 | 0 | 1081 | 2652 | 0 | 1083 | 2650 | 0 | 1079 | 2642 | 0 | 1079 | 2648 | 0 | 1080 | 2644 | 0 |
| *trnL2* | 2656 | 2730 | 2 | 2653 | 2727 | 2 | 2651 | 2725 | 2 | 2643 | 2717 | 2 | 2649 | 2723 | 2 | 2645 | 2719 | 2 |
| *nad1* | 2733 | 3689 | -2 | 2730 | 3686 | -2 | 2728 | 3682 | 0 | 2720 | 3674 | 0 | 2726 | 3680 | 0 | 2722 | 3676 | 0 |
| *trnI* | 3688 | 3756 | -3 | 3685 | 3753 | -3 | 3683 | 3751 | -3 | 3675 | 3743 | -3 | 3681 | 3749 | -3 | 3677 | 3745 | -3 |
| *trnQ* | 3754 | 3824 | -1 | 3751 | 3821 | -1 | 3749 | 3819 | -1 | 3741 | 3811 | -1 | 3747 | 3817 | -1 | 3743 | 3813 | -1 |
| *trnM* | 3824 | 3892 | 0 | 3821 | 3889 | 0 | 3819 | 3887 | 0 | 3811 | 3879 | 0 | 3817 | 3885 | 0 | 3813 | 3881 | 0 |
| *nad2* | 3893 | 4936 | -2 | 3890 | 4933 | -2 | 3888 | 4929 | 0 | 3880 | 4921 | 0 | 3886 | 4927 | 0 | 3882 | 4923 | 0 |
| *trnW* | 4935 | 5001 | 4 | 4932 | 4999 | 5 | 4930 | 4997 | 4 | 4922 | 4988 | 4 | 4928 | 4994 | 4 | 4924 | 4990 | 4 |
| *trnA* | 5006 | 5075 | 1 | 5005 | 5074 | 1 | 5002 | 5071 | 1 | 4993 | 5062 | 1 | 4999 | 5068 | 1 | 4995 | 5064 | 1 |
| *trnN* | 5077 | 5149 | 33 | 5076 | 5148 | 33 | 5073 | 5145 | 33 | 5064 | 5136 | 33 | 5070 | 5142 | 33 | 5066 | 5138 | 33 |
| *trnC* | 5183 | 5249 | 0 | 5182 | 5249 | 0 | 5179 | 5245 | 0 | 5170 | 5236 | 0 | 5176 | 5242 | 0 | 5172 | 5239 | 0 |
| *trnY* | 5250 | 5315 | 1 | 5250 | 5316 | 0 | 5246 | 5312 | 0 | 5237 | 5303 | 0 | 5243 | 5309 | 0 | 5240 | 5306 | 0 |
| *cox1* | 5317 | 6864 | -5 | 5317 | 6858 | 2 | 5313 | 6854 | 1 | 5304 | 6845 | 1 | 5310 | 6851 | 1 | 5307 | 6848 | 1 |
| *trnS2* | 6860 | 6928 | 4 | 6861 | 6929 | 4 | 6856 | 6924 | 4 | 6847 | 6915 | 4 | 6853 | 6921 | 4 | 6850 | 6918 | 4 |
| *trnD* | 6933 | 7001 | 0 | 6934 | 7002 | 0 | 6929 | 6997 | 0 | 6920 | 6988 | 0 | 6926 | 6994 | 0 | 6923 | 6991 | 0 |
| *cox2* | 7002 | 7685 | 1 | 7003 | 7686 | 0 | 6998 | 7681 | 1 | 6989 | 7672 | 1 | 6995 | 7678 | 1 | 6992 | 7675 | 1 |
| *trnK* | 7687 | 7750 | 2 | 7687 | 7752 | 2 | 7683 | 7745 | 2 | 7674 | 7737 | 2 | 7680 | 7743 | 2 | 7677 | 7740 | 2 |
| *atp8* | 7753 | 7956 | -43 | 7755 | 7958 | -43 | 7748 | 7951 | -43 | 7740 | 7943 | -43 | 7746 | 7949 | -43 | 7743 | 7946 | -43 |
| *atp6* | 7914 | 8594 | -1 | 7916 | 8596 | -1 | 7909 | 8589 | -1 | 7901 | 8581 | -1 | 7907 | 8587 | -1 | 7904 | 8584 | -1 |
| *cox3* | 8594 | 9379 | -1 | 8596 | 9381 | -1 | 8589 | 9373 | 0 | 8581 | 9365 | 0 | 8587 | 9371 | 0 | 8584 | 9368 | 0 |
| *trnG* | 9379 | 9445 | 9 | 9381 | 9446 | 0 | 9374 | 9438 | 0 | 9366 | 9431 | 0 | 9372 | 9437 | 0 | 9369 | 9435 | 0 |
| *nad3* | 9455 | 9802 | -10 | 9447 | 9793 | 0 | 9439 | 9785 | 0 | 9432 | 9778 | 0 | 9438 | 9784 | 0 | 9436 | 9782 | 0 |
| *trnR* | 9793 | 9858 | 1 | 9794 | 9859 | 1 | 9786 | 9851 | 1 | 9779 | 9844 | 1 | 9785 | 9850 | 1 | 9783 | 9848 | 1 |
| *nad4l* | 9860 | 10156 | -7 | 9861 | 10157 | -7 | 9853 | 10149 | -7 | 9846 | 10142 | -7 | 9852 | 10148 | -7 | 9850 | 10146 | -7 |
| *nad4* | 10150 | 11527 | 0 | 10151 | 11528 | 0 | 10143 | 11520 | 0 | 10136 | 11513 | 0 | 10142 | 11519 | 0 | 10140 | 11517 | 0 |
| *trnH* | 11528 | 11596 | 0 | 11529 | 11597 | 0 | 11521 | 11589 | 0 | 11514 | 11582 | 0 | 11520 | 11588 | 0 | 11518 | 11586 | 0 |
| *trnS1* | 11597 | 11655 | -1 | 11598 | 11655 | -1 | 11590 | 11649 | -1 | 11583 | 11642 | -1 | 11589 | 11648 | -1 | 11587 | 11646 | -1 |
| *trnL1* | 11655 | 11724 | -9 | 11655 | 11724 | 0 | 11649 | 11718 | 0 | 11642 | 11711 | 0 | 11648 | 11717 | 0 | 11646 | 11715 | 0 |
| *nad5* | 11716 | 13536 | 2 | 11725 | 13536 | 1 | 11719 | 13530 | 2 | 11712 | 13523 | 2 | 11718 | 13529 | 2 | 11716 | 13527 | 2 |
| *nad6* | 13539 | 14060 | 0 | 13538 | 14059 | 0 | 13533 | 14054 | 0 | 13526 | 14047 | 0 | 13532 | 14053 | 0 | 13530 | 14051 | 0 |
| *trnE* | 14061 | 14128 | 3 | 14060 | 14127 | 3 | 14055 | 14121 | 3 | 14048 | 14115 | 3 | 14054 | 14121 | 3 | 14052 | 14119 | 3 |
| *cob* | 14132 | 15271 | -1 | 14131 | 15270 | 0 | 14125 | 15264 | 0 | 14119 | 15258 | 0 | 14125 | 15264 | 0 | 14123 | 15262 | 0 |
| *trnT* | 15271 | 15338 | 1 | 15271 | 15335 | 2 | 15265 | 15329 | 2 | 15259 | 15323 | 2 | 15265 | 15329 | 2 | 15263 | 15327 | 2 |
| *trnP* | 15340 | 15407 | 0 | 15338 | 15404 | 0 | 15332 | 15399 | 0 | 15326 | 15393 | 0 | 15332 | 15399 | 0 | 15330 | 15397 | 0 |
| CR | 15408 | 17164 |  | 15405 | 16754 |  | 15400 | 16187 |  | 15394 | 16183 |  | 15400 | 16189 |  | 15398 | 16186 |  |

**Table S4.** Start and Stop codon of PCGs among six Tupaiidae species.

| **PCGs** | ***T. nicobarica*** | | ***T. belangeri*** | | ***T. minor*** | | ***T. montana*** | | ***T. splendidula*** | | ***T. tana*** | |
| --- | --- | --- | --- | --- | --- | --- | --- | --- | --- | --- | --- | --- |
|  | Start | Stop | Start | Stop | Start | Stop | Start | Stop | Start | Stop | Start | Stop |
| *nad1* | ATG | TAG | ATG | TAG | ATG | CAT | ATG | CAT | ATG | CAT | ATG | CAT |
| *nad2* | ATC | TAG | ATC | TAG | ATC | TCT | ATT | TTT | ATT | TTT | ATT | TTT |
| *cox1* | ATG | AGA | ATG | TAA | ATG | TAG | ATG | TAG | ATG | TAG | ATG | TAG |
| *cox2* | ATG | TAG | ATG | TAG | ATG | TAA | ATG | TAA | ATG | TAA | ATG | TAA |
| *atp8* | ATG | TAA | ATG | TAA | ATG | TAA | ATG | TAA | ATG | TAA | ATG | TAA |
| *atp6* | ATG | TAA | ATG | TAA | ATG | TAA | ATG | TAA | ATG | TAA | ATG | TAA |
| *cox3* | ATG | TAA | ATG | TAA | ATG | ATA | ATG | ATA | ATG | ATA | ATG | TTA |
| *nad3* | ATT | TAG | ATA | ATA | ATT | ATA | ATA | ATA | ATA | ATA | ATA | ATA |
| *nad4l* | ATG | TAA | ATG | TAA | ATG | TAA | ATG | TAA | ATG | TAA | ATG | TAA |
| *nad4* | ATG | T(AA) | ATG | TAT | ATG | TAT | ATG | TAT | ATG | TAT | ATG | TAT |
| *nad5* | ATA | TAG | ATC | TAA | ATT | TAA | ATT | TAA | ATT | TAG | ATT | TAA |
| *nad6* | ATG | AGG | ATG | TAG | ATG | AGG | ATG | AGA | ATG | AGG | ATG | AGA |
| *cytb* | ATG | TAG | ATG | TAG | ATG | TAG | ATG | TAG | ATG | TAG | ATG | TAG |

**Table S5.** Pairwise divergence of non-synonymous (Ka) and synonymous (Ks) substitutions ratio among the six Tupaiidae species.

| Species | Species | ***atp6*** | ***atp8*** | ***cox1*** | ***cox2*** | ***cox3*** | ***cytb*** | ***nad1*** | ***nad2*** | ***nad3*** | ***nad4*** | ***nad4l*** | ***nad5*** | ***nad6*** |
| --- | --- | --- | --- | --- | --- | --- | --- | --- | --- | --- | --- | --- | --- | --- |
| *T. belangeri* | *T. minor* | 0.031 | 0.229 | 0.006 | 0.012 | 0.014 | 0.033 | 0.034 | 0.146 | 0.038 | 0.039 | 0.038 | 0.075 | 0.069 |
| *T. belangeri* | *T. montana* | 0.022 | 0.183 | 0.006 | 0.017 | 0.020 | 0.035 | 0.050 | 0.118 | 0.009 | 0.042 | 0.042 | 0.084 | 0.045 |
| *T. belangeri* | *T. nicobarica* | 0.038 | 0.231 | 0.008 | 0.019 | 0.012 | 0.042 | 0.024 | 0.127 | 0.034 | 0.050 | 0.029 | 0.070 | 0.054 |
| *T. belangeri* | *T. splendidula* | 0.026 | 0.250 | 0.006 | 0.014 | 0.017 | 0.033 | 0.039 | 0.104 | 0.018 | 0.050 | 0.046 | 0.071 | 0.064 |
| *T. belangeri* | *T. tana* | 0.029 | 0.146 | 0.005 | 0.015 | 0.018 | 0.026 | 0.032 | 0.111 | 0.020 | 0.051 | 0.052 | 0.070 | 0.052 |
| *T. minor* | *T. montana* | 0.022 | 0.105 | 0.002 | 0.004 | 0.005 | 0.027 | 0.035 | 0.078 | 0.024 | 0.026 | 0.066 | 0.060 | 0.025 |
| *T. minor* | *T. nicobarica* | 0.025 | 0.119 | 0.005 | 0.017 | 0.008 | 0.041 | 0.040 | 0.061 | 0.024 | 0.029 | 0.034 | 0.059 | 0.036 |
| *T. minor* | *T. splendidula* | 0.020 | 0.310 | 0.004 | 0.012 | 0.005 | 0.023 | 0.038 | 0.069 | 0.025 | 0.026 | 0.036 | 0.053 | 0.031 |
| *T. minor* | *T. tana* | 0.020 | 0.114 | 0.005 | 0.011 | 0.014 | 0.026 | 0.041 | 0.053 | 0.021 | 0.035 | 0.054 | 0.064 | 0.021 |
| *T. montana* | *T. nicobarica* | 0.014 | 0.127 | 0.008 | 0.017 | 0.012 | 0.035 | 0.046 | 0.076 | 0.031 | 0.031 | 0.030 | 0.075 | 0.037 |
| *T. montana* | *T. splendidula* | 0.020 | 0.051 | 0.004 | 0.012 | 0.007 | 0.030 | 0.025 | 0.042 | 0.050 | 0.032 | 0.060 | 0.056 | 0.044 |
| *T. montana* | *T. tana* | 0.022 | 0.076 | 0.006 | 0.016 | 0.020 | 0.025 | 0.020 | 0.043 | 0.018 | 0.036 | 0.050 | 0.057 | 0.043 |
| *T. nicobarica* | *T. splendidula* | 0.019 | 0.176 | 0.007 | 0.013 | 0.010 | 0.031 | 0.042 | 0.075 | 0.023 | 0.036 | 0.036 | 0.074 | 0.029 |
| *T. nicobarica* | *T. tana* | 0.026 | 0.078 | 0.010 | 0.022 | 0.013 | 0.032 | 0.046 | 0.069 | 0.030 | 0.030 | 0.045 | 0.056 | 0.031 |
| *T. splendidula* | *T. tana* | 0.024 | 0.109 | 0.007 | 0.022 | 0.016 | 0.022 | 0.024 | 0.037 | 0.023 | 0.027 | 0.056 | 0.055 | 0.047 |

**Figure S1.** Box plot for the pairwise divergence of non-synonymous and synonymous substitutions ratio among six Tupaiidae species.


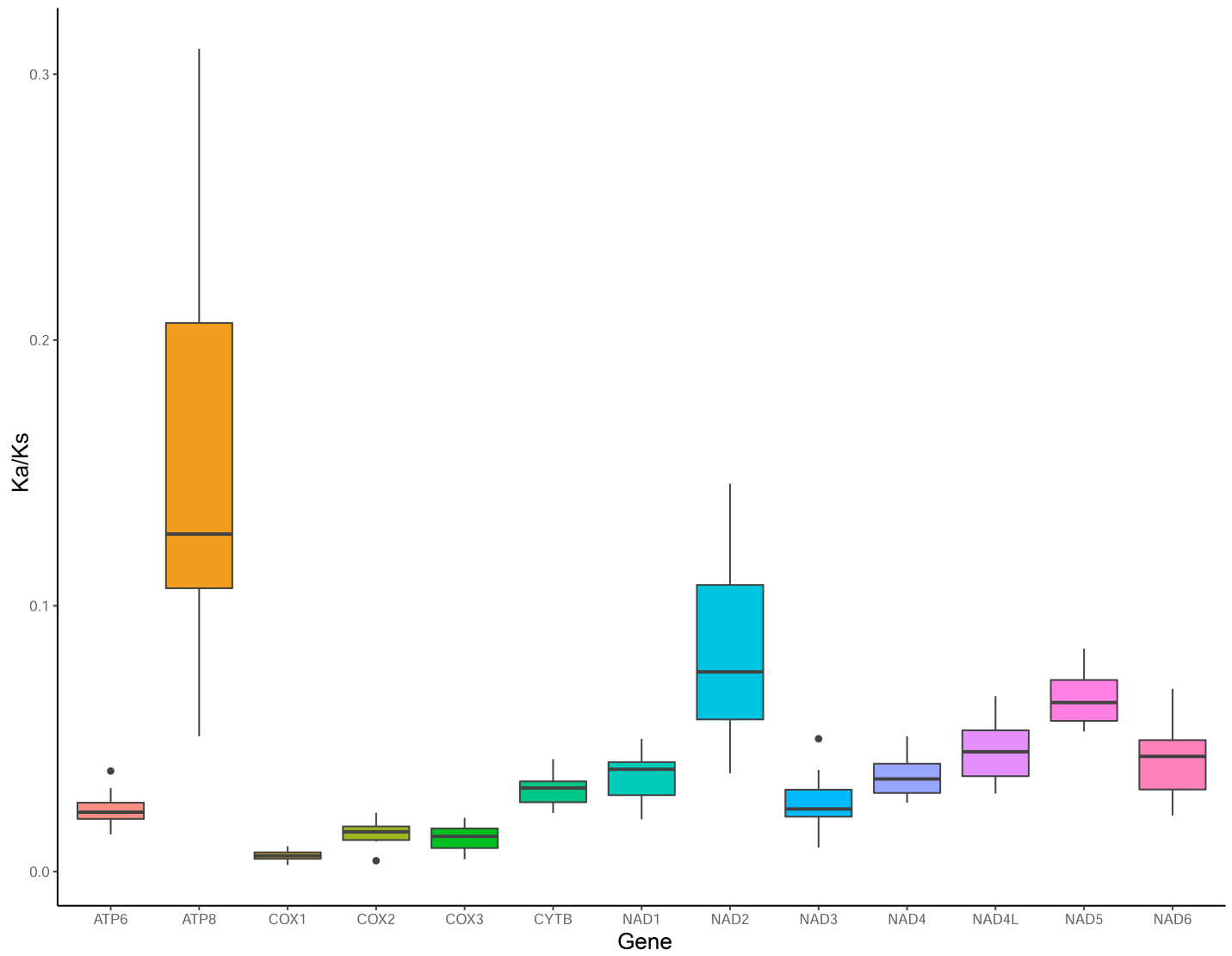


**Figure S2.** Comparative Relative Synonymous Codon Wsage (RSCU) in six Tupaiidae species,
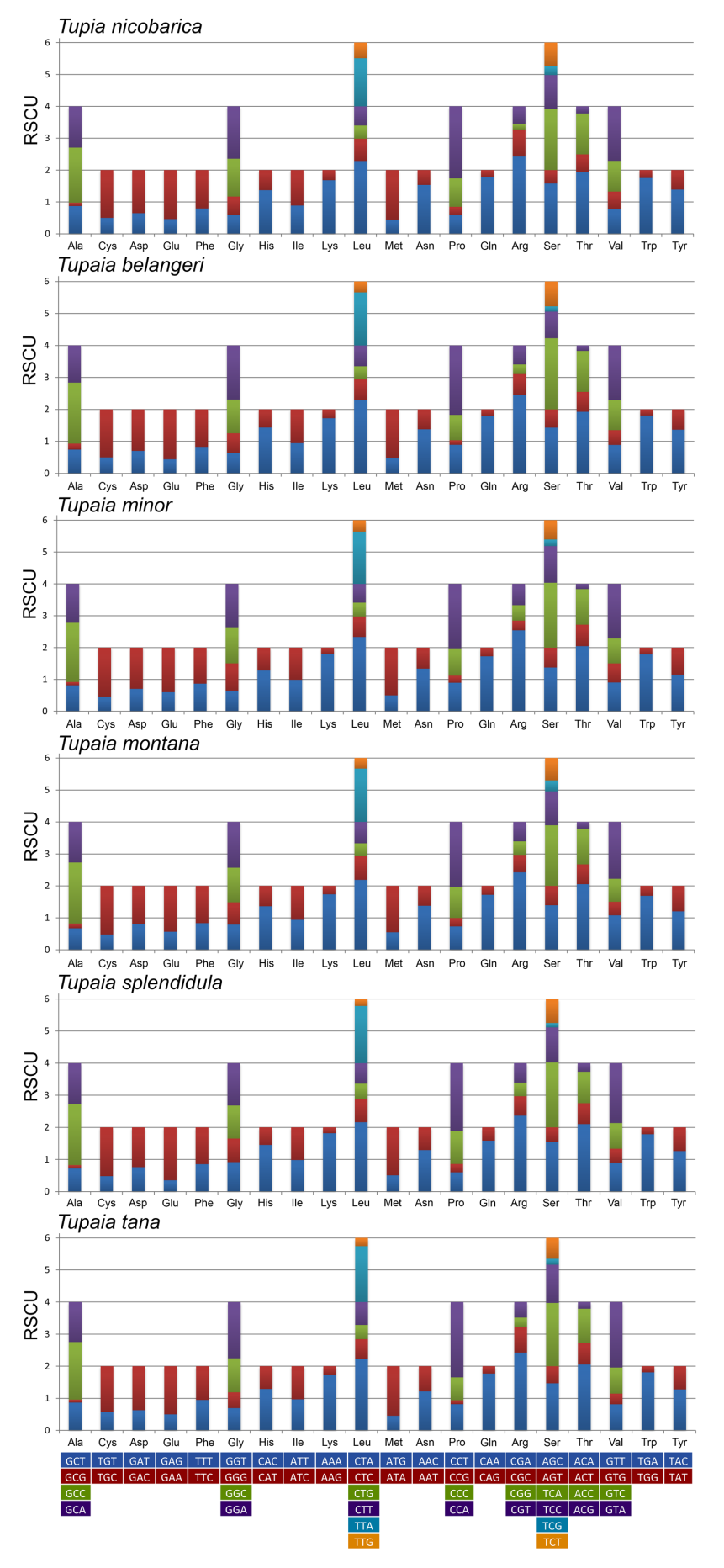
including *T. nicobarica*.

**Figure S3.** Secondary structures of 22 transfer RNAs (tRNAs) exhibiting the structural variation in *T. nicobarica* mitogenome. The first structure shows the nucleotide positions and details of the stem-loop of tRNAs. The tRNAs are represented by full names and IUPAC-IUB single-letter amino acid codes.

**
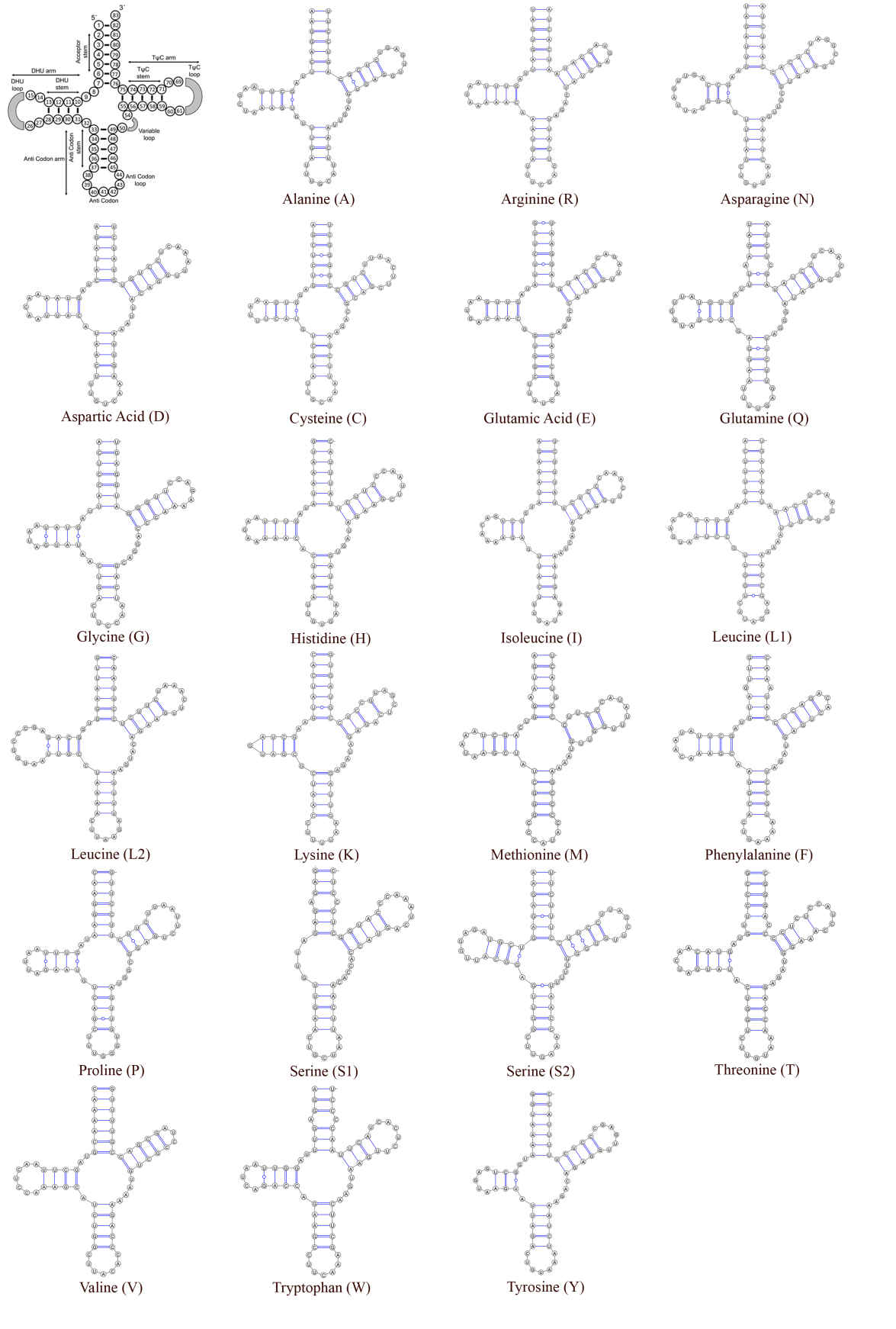
**

**Figure S4.** Maximum-Likelihood phylogeny of six Tupaiidae species inferred by concatenated PCGs showed distinct clustering of *T. nicobarica*.


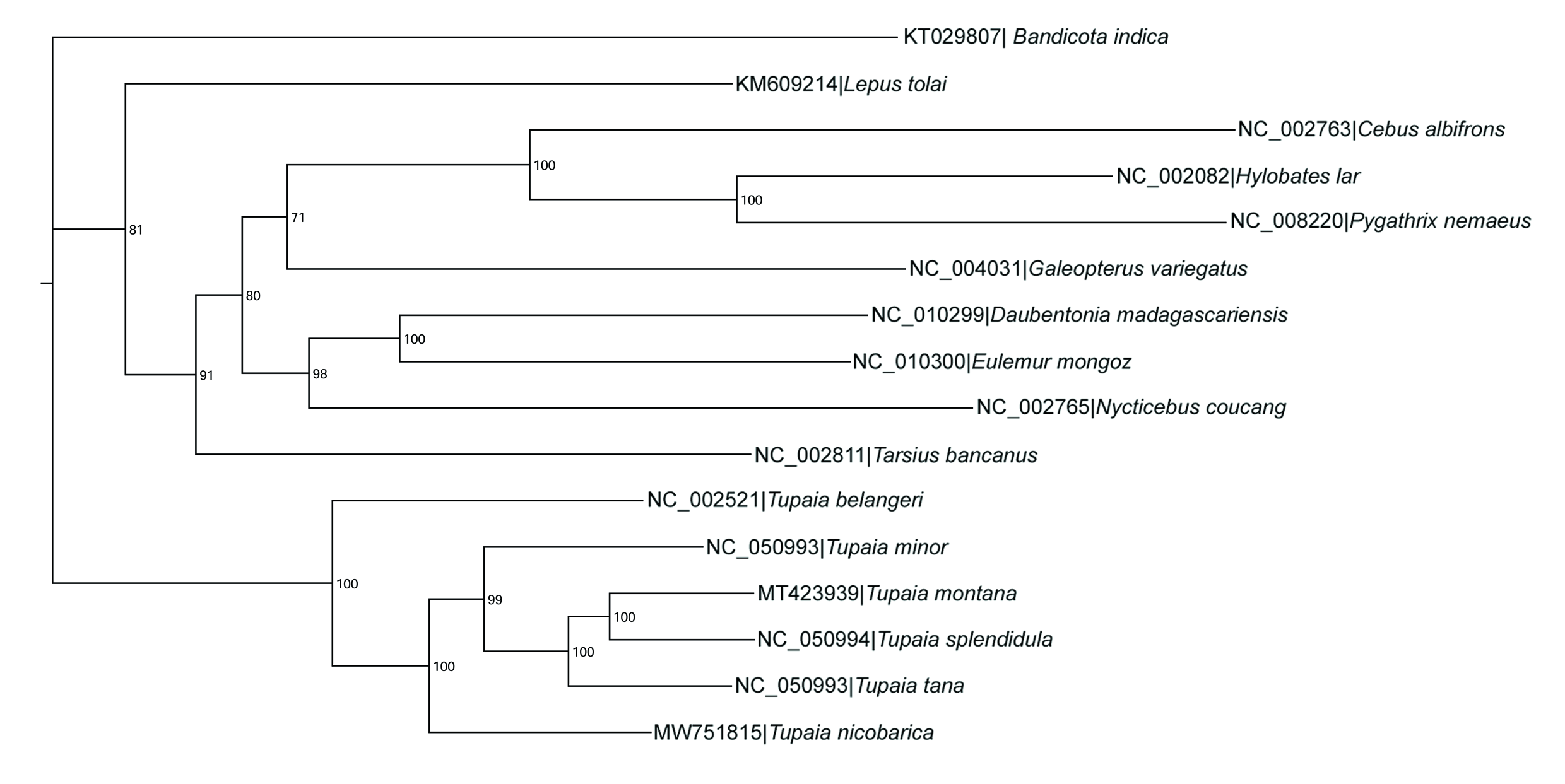

Supplement: Supplementary file 1 — Supplementary Information. [file 41598_2022_4907_MOESM1_ESM.docx]
